# Abnormal developmental trends of functional connectivity in young children with infantile esotropia

**DOI:** 10.3389/fnins.2022.972882

**Published:** 2022-08-18

**Authors:** Jianlin Guo, Yuanyuan Chen, Wen Liu, Lijuan Huang, Di Hu, Yanqiu Lv, Huiying Kang, Ningdong Li, Yun Peng

**Affiliations:** ^1^Department of Radiology, Beijing Children’s Hospital, Capital Medical University, National Center for Children’s Health, Beijing, China; ^2^Tianjin International Joint Research Center for Neural Engineering, Academy of Medical Engineering and Translational Medicine, Tianjin University, Tianjin, China; ^3^Department of Ophthalmology, Beijing Children’s Hospital, Capital Medical University, National Center for Children’s Health, Beijing, China; ^4^Department of Ophthalmology, The Second Affiliated Hospital of Fujian Medical University, Quanzhou, China; ^5^Key Laboratory of Major Diseases in Children, Ministry of Education, Beijing, China

**Keywords:** infantile esotropia, functional connectivity, development, functional magnetic resonance imaging, children

## Abstract

Previous studies have shown that functional networks are present at birth and change dynamically throughout infancy and early childhood. However, the status of functional connectivity is still poorly understood in patients with infantile esotropia (IE). The aim of this study is to investigate the developmental trends of functional connectivity in patients with IE during a critical period of growth and development. A total of 17 patients with IE (9 males and 8 females; mean age: 3.36 ± 2.03 years, age range: 0.67–6.36 years) and 20 healthy subjects matched for age and gender were recruited and underwent resting-state functional magnetic resonance imaging. The whole-brain functional network connectivity was analyzed for the IE group and healthy control group. A general linear model was applied to assess the group-age interaction in terms of the functional connectivity. The discrepancy between the two groups in functional connectivity trajectories was also quantified across age and exhibited by the quadratic parabolic model. There were significant group-age interactions between the visual network and the default mode network, the visual network and the sensorimotor network, the limbic network and the default mode network, and within the limbic network in the functional connectivity. A U-shaped tendency across age, with an “inflection point” ranging from 3.1 to 4.0 years of age was exhibited in the difference between functional connectivity trajectories of the IE patients and normal controls. Abnormality in functional network connectivity could present in IE patients at birth, exhibiting aberrant developmental patterns over time. An abnormal functional network could reduce the ability of the cortex in visual information processing, further reactivating the subcortical visual information processing system, which is probably the pathogenesis of IE. Three to four years after birth is the critical time window for children with IE to establish normal network connections in the brain. Early surgery during this period may be helpful for affected children to have an opportunity to approach the normal development trajectory as early as possible.

## Introduction

Infantile esotropia (IE) or congenital esotropia, refers to an inward deviation of eyes within the early 6 months of life, with an estimated incidence of 0.1–1% in the population (Magli et al., 2017). It is characterized as an alternate esotropia with a large deviation angle, and usually associated with oblique muscle dysfunction, dissociated vertical deviation (DVD), and latent nystagmus (Brodsky, 2019). Many affected children may have abnormal pursuit movement and asymmetry of the monocular optokinetic response, presenting as a congenital deficiency of binocular single vision which could be probably caused by a defect in sensory fusion mechanism or by defect in motor fusion mechanism. A recent study shows that IE is caused by perturbation of binocularity development in the striate cortex within the early 3 months of life, which results in re-activation of monocularly driven subcortical optokinetic pathways that allow an early subcortical nasalward optokinetic bias to drive the eyes into inward position (Brodsky, 2018). However, the pathogenesis of IE is still not clear in detail.

Surgical treatment is the only choice for IE management. However, the time of surgery is debatable. Opinions in favor of early surgery are: (1) helpful for establishing binocular vision and stereopsis as early as possible and minimizing developmental delay of sensorimotor and gross motor; (2) effective in reducing incidence and severity of postoperative DVD and inferior oblique overaction; (3) beneficial for improvement of cosmetic appearance and self-confidence, although accurate evaluation of deviation angle for an infant is challenging. On the contrary, opinions in favor of late surgery are based on the fact that the old children are able to cooperate very well in measurement of the deviation angles both at near and distance, and in estimating ocular movement so that an accurate preoperative evaluation could be achieved.

Previous studies have shown that the functional networks in the brain have been present prenatally and change dynamically throughout infancy and early childhood, with differential developmental trajectories with age (Gao et al., 2017; Marrus et al., 2018; Bruchhage et al., 2020). The functional networks in the brain interact flexibly with each other to meet the needs of a variety of complex abilities. During the development of the visual perception system, it constantly interacts with the ocular movement efferent system to construct a perfect sensorimotor network (SMN) connectivity to meet the needs of the cognitive world. In addition, the core regions of visual network (VN) have widespread functional connections with the limbic network (LN), involved in high-order cognitive and emotional processing (Panitz et al., 2021). The motor regions of the brain also improve their functional connections with the VN to achieve “hand-eye” coordination as they develop (Huang and Barber, 2021).

During the critical period of cerebral cortical maturation, coordinated input from each eye to visual cortex is a pre-requisite for VN development, while impaired binocular vision due to misalignment of both eyes would lead to a neurodevelopmental disorder of vision (Thompson et al., 2015). Abnormal functional network connections are detected in patients with concomitant exotropia who exhibit reduced functional connectivity of the V1 with lingual gyrus, precentral gyrus and inferior parietal lobule (Zhu et al., 2018). Moreover, enhanced functional connectivity is found between the V1 and such cortical areas as extrastriate visual cortex and frontal eye field in patients with concomitant strabismus (Yan et al., 2019). These findings demonstrate significant brain functional connectivity changes in patients with strabismus, which are related to impaired binocular vision. However, as a congenital sensory defect strabismus, it is not clear about the development of the visual functional connections under the influence of IE. Therefore, the purpose of this study is to explore the functional network connections in the patients with IE.

## Materials and methods

### Participants

A total of 17 patients with IE were recruited in this study, including 9 males and 8 females with a mean age of 3.36 ± 2.03 (range from 0.67 to 6.36 years). Diagnostic criteria for IE were based on the onset of eso-deviation within the age of 6 months, and alternate esotropia with a deviation angle of 40–70 prism diopters without limitation of ocular movement at any gaze positions. All children were full-term pregnancy and normal delivery without mental and neurological disorders. Twenty age- and sex-matched healthy individuals were included as normal controls. This study was approved by the Medical Research Ethics Committee of Beijing Children’s Hospital and complied with the tenets of the Declaration of Helsinki. The written informed consent was obtained from the parents or legal guardians of the subjects.

### MRI parameters

Imaging data was collected using a 3.0-T GE DISCOVERY MR750 scanner (General Electric, Milwaukee, United States) with an eight-channel head coil. All subjects were scanned during natural sleep, in order to avoid mixture of functional MRI (fMRI) recordings under sleep status with the recordings under awake condition. All participants were scheduled at night when they felt sleepy and examined in a supine position with a headphone to protect noise intrusion and a foam pad to prevent head movement.

T1-weighted, sagittal 3D brain volume (BRAVO) sequence was acquired with the following parameters: repetition time/echo time = 8.2 ms/3.2 ms, flip angle = 12°, field of view = 256 mm × 256 mm, acquisition matrix = 256 × 256, voxel size = 1 mm × 1 mm × 1 mm, slice = 164, slice thickness = 1 mm, and no gap. Resting-state fMRI data were obtained with an echo-planar imaging sequence and the scanning parameters were as follows: repetition time/echo time = 2000 ms/30 ms, field of view = 224 mm × 224 mm, matrix = 64 × 64, flip angle = 90°, slice thickness = 3.5 mm, slice gap = 0 mm, slice = 40, voxel size = 3.5 mm × 3.5 mm × 3.5 mm, and 240 volumes in total.

### Functional MRI data pre-processing

The fMRI images were preprocessed using the programs of Data Processing Assistant for Resting-State fMRI (Yan et al., 2016) in MATLAB2020b (Mathworks, Natick, MA, United States). The raw data of Digital Imaging and Communications in Medicine (DICOM) files were converted into NIFTI images for subsequent analysis. Considering that the MRI signal may be unstable at the beginning, the data acquired at the first 10 time points were discarded. For the remaining 230 images, bias correction was performed to eliminate the time difference between slices and to correct the head motion during the scanning process. Images were discarded if the head was translated more than 1.5 mm at any direction of *x*, *y*, or *z* axis, and rotated more than 1.5°. The interference of head movement, whole brain signals, white matter signals and cerebral fluid signals on image analysis was eliminated through nuisance covariates regression. The de-liner drift was applied to reduce the interferences of the linear drift related to the long duration of the scan. A bandpass filter with a frequency range of 0.01–0.1 Hz was used to eliminate the influence of low-frequency drift and high-frequency noise caused by heartbeat and breathing. The data was spatially smoothed with a Gaussian kernel of 4 mm full width at half maximum to remove spatial noise. The functional images were spatially normalized to the infant brain template through co-registration using University of North Carolina (UNC) infant brain template (Shi et al., 2011) and warping to the age-specific template using Advanced Normalization Tools (ANTs) (Avants et al., 2014), which has been proven to be effective for registering pediatric data (Liu et al., 2021), in order to overcome the difference of brain structure and volume among each subject. All subjects were checked visually to ensure the quality of the registration.

### Construction of whole-brain functional network

A total of 90 (45 in each hemisphere) regions of interest (ROIs) were parcellated according to the UNC infant brain atlas (Shi et al., 2011), which was anatomically consistent with the Automated Anatomical Labeling (AAL) atlas (Tzourio-Mazoyer et al., 2002), and listed in Supplementary Table 1. The average temporal series of all voxels within each anatomical ROI was extracted in standard space. The correlation of temporal series between each ROI pairs was calculated and Fisher z-transformed for subsequent statistical analysis.

### Statistical analysis

To examine the difference between IE patients and the normal controls in brain functional connectivity development, the general linear model (GLM) was applied to evaluate the interactive effects of group (IE patients vs. normal controls) with age in functional connectivity, with independent variables of age, gender, group, age × gender, age × group, and gender × group. Due to the exploratory nature of this study, a *P*-value of 0.001 was set up as a significant threshold instead of multiple comparison correction. Partial correlation analysis was used to identify the developmental trends in functional connectivity with the age-group interactions in the IE group and in the control group, respectively, with correction of gender. A *P*-value of < 0.05 was considered statistically significant. The group differences of functional connectivity were also quantified across age based on GLM and exhibited by the quadratic parabolic model. Statistical analysis was performed using the tool of SPSS 23.0 (SPSS Inc., Chicago, IL, United States) for clinical and demographic data. An independent *t*-test was used to compare the continuous data between IE and normal groups. An exact Fisher test was used to compare the categorical data in the two groups mentioned above. A *P*-value of < 0.05 was considered statistically significant.

## Results

### Demographical and clinical features

The demographics and clinical characteristics of the two groups were listed in Table 1. Group differences in age and gender were examined using two-sample two-tailed *t*-test and Fisher’s exact test. No statistical difference was found in age (*P* = 0.932) or gender (*P* > 0.99) between the two groups (Table 1).

**TABLE 1:** Demographical and clinical features of study population.

	IEs (*n* = 17)	HCs (*n* = 20)	*P*-value
Age (years)	3.36 ± 2.03	3.41 ± 1.57	0.932*
Sex, male/female	9/8	11/9	>0.99^#^
Onset age (months)	4.06 ± 1.92	N/A	N/A
Duration (months)	36.06 ± 23.61	N/A	N/A
Angle of strabismus (PD)	51.47 ± 20.22	N/A	N/A

IEs, infantile esotropia patients; HCs, healthy controls; N/A, not applicable; PD, prism degree.

*Two-sample two-tailed t-test.

^#^Fisher’s exact test.

### Functional connections with significant group-age interaction

**FIGURE 1:**
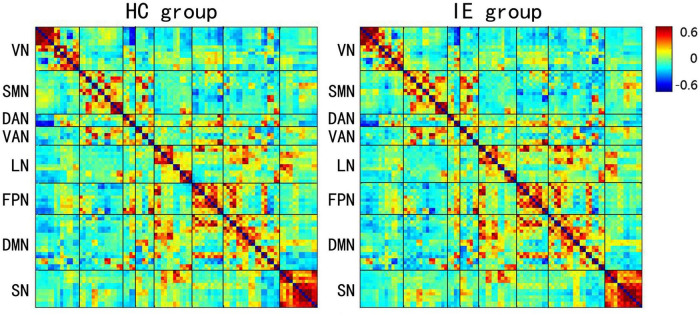
Mean *Z*-score Matrices for Healthy Subjects and Patients with Infantile Esotropia. Each figure exhibits a 90 × 90 square matrix. The colored bar indicates the *z*-score of the functional connectivity. HC, healthy control; IE, infantile esotropia; VN, visual network; SMN, sensorimotor network; DAN, dorsal attention network; VAN, ventral attention network; LN, limbic network; FPN, frontoparietal network; DMN, default mode network; SN, subcortical network.

**FIGURE 2:**
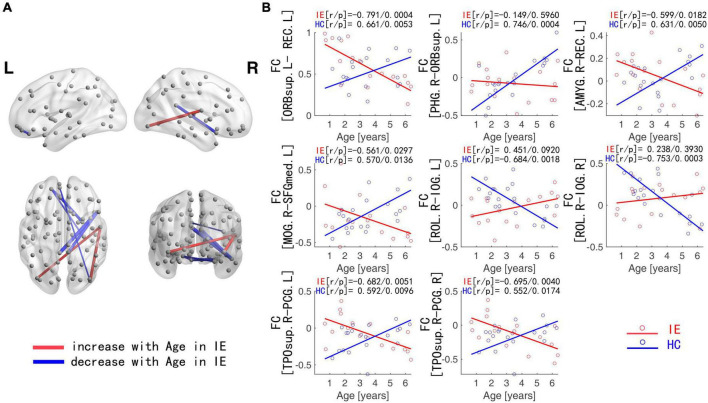
Functional Connections with Significant Interaction between Group and Age. **(A)** Blue edges: functional connections with the z-score declining with age in IE patients; Red edges: functional connections with the *z*-score increasing with age in IE patients. **(B)** Age-dependent decreased functional connectivity was observed in ORBsup.L-REC.L, PHG.R-ORBsup.L, AMYG.R-REC.L, MOG.R-SFGmed.L, and PCG-TPOsup.R in IE patients in contrast to the increase for HCs, whereas increased functional connectivity with age was found in ROL.R-IOG in IE patients in contrast to the decline for HCs. IE, infantile esotropia; HC, healthy control; L, left; R, right; ORBsup, orbital part of superior frontal gyrus; REC, gyrus rectus; PHG, parahippocampal gyrus; AMYG, amygdala; MOG, middle occipital gyrus; SFGmed, superior frontal gyrus, medial; PCG, posterior cingulate gyrus; TPOsup, temporal pole of superior temporal gyrus; ROL, Rolandic operculum; IOG, inferior occipital gyrus.

The connectivity matrices in whole-brain network were constructed in IE patients and normal controls, respectively (Figure 1). A significant group-age interaction was found in the functional connectivity between the VN and the following networks of the default mode network (DMN) and the SMN, between the LN and DMN, as well as within LN itself. In addition, a significant group-age interaction was exhibited in the functional connectivity in multiple areas in the brain, such as functional connections of left gyrus rectus (REC) with both left orbital part of superior frontal gyrus (ORBsup) and right amygdala (AMYG); of right parahippocampal gyrus (PHG) and left ORBsup; of right middle occipital gyrus (MOG) and left medial superior frontal gyrus (SFGmed); of posterior cingulate gyrus (PCG) and right temporal lobe (temporal pole of superior temporal gyrus, TPOsup); of inferior occipital gyrus (IOG) and right Rolandic operculum (ROL) (*P* < 0.001) (Table 2). In contrast to the increase of functional connectivity in the normal control group, there was a declined trend of the functional connectivity with age between left ORBsup and left REC, right AMYG and left REC, right MOG and left SFGmed, bilateral PCG and right TPOsup in the IE group (*P* < 0.05) (Figure 2). There was a positive correlation of right PHG and left ORBsup with age in functional connectivity in the control group, and a negative correlation of right ROL and bilateral IOG with age (*P* < 0.05). However, there were no correlations of functional connections with age at either right PHG and left ORBsup with age, or right ROL and bilateral IOG in the IE group (*P* > 0.05) (Figure 2).

**TABLE 2:** Functional connections with significant group-age interactions.

Functional connection	*T*-value	*P*-value (10^–3^)
**LN-LN**		
L orbital part of superior frontal gyrus-L gyrus rectus	–3.96	0.428
R parahippocampal gyrus–L orbital part of superior frontal gyrus	–3.77	0.706
R amygdala-L gyrus rectus	–4.15	0.252
**VN-DMN**		
R middle occipital gyrus-L superior frontal gyrus, medial	–3.70	0.870
**DMN-LN**		
L posterior cingulate gyrus-R temporal pole: superior temporal gyrus	–4.40	0.128
R posterior cingulate gyrus-R temporal pole: superior temporal gyrus	–4.22	0.208
**SMN-VN**		
R Rolandic operculum-L inferior occipital gyrus	4.04	0.344
R Rolandic operculum-R inferior occipital gyrus	4.19	0.224

LN, limbic network; VN, visual network; DMN, default mode network; SMN, sensorimotor network; L, left; R, right.

### Group differences of functional connectivity trajectory across age

**FIGURE 3:**
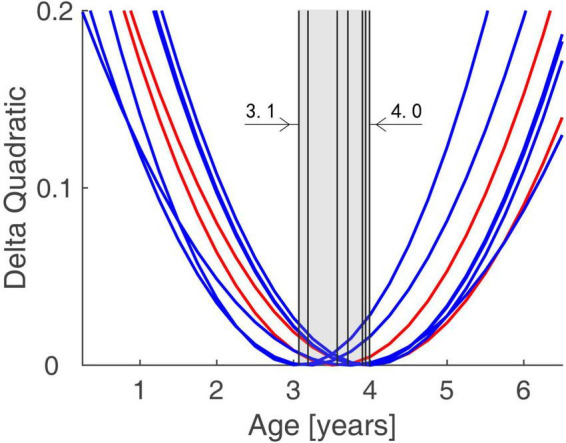
Trends of Group Differences in terms of Functional Connectivity Trajectory. The difference between the trajectories of two groups formed a U-shaped curve with the age trajectory, with an inflection point at the age of 3.1–4.0.

The functional connectivity developed linearly with age in both normal subjects and IE patients. However, they had their own developmental trajectories. The difference between their trajectories formed a U-shaped curve with the age trajectory, with an inflection point at the age of 3–4. This U-shaped tendency across age was detected in the functional connectivity between the following areas of left ORBsup and left REC, right PHG and left ORBsup, right AMYG and left REC, right MOG and left SFGmed, bilateral PCG and right TPOsup, right ROL and bilateral IOG (Figure 3).

## Discussion

In this study, we compare the developmental trends of whole-brain functional connectivity in IE patients with that in normal controls, and find that the IE patients have aberrant functional connectivity in the intra- and inter-networks, including the network connectivity of LN-LN, VN-DMN, DMN-LN, and VN-SMN, which could present very early after birth. The functional connectivity network has different developmental trajectory in IE patients and control controls, characterized as developmental trajectory separating at the beginning of life, converging around the age of 3–4 years and then separating again over time (Figure 2). The difference between their trajectories formed a U-shaped curve with the age trajectory, with an inflection point at the age of 3–4 (Figure 3).

Patients with IE look at the world using their abnormal binocular vision, so that they have to establish the corresponding functional network connectivity in their brain to adapt to the environment they are living in. Because we do not know the exact etiology of esotropia, we do not know when they begin to establish such functional connectivity in their brain. However, we find that they have their own development trajectory in functional connectivity. Although the starting point of the trajectory is different at the beginning of life, their trajectory has a tendency to approach those of normal controls. At the period of 3–4 years, their functional connectivity is similar to those of normal controls and approaches each other. After this period, their functional connectivity will once again separate from the normal trajectory and become further and further apart from each other. Thus, it is reasonable to speculate that the period before the age of 3–4 is a critical period for the IE patients to build a normal functional connectivity in their brain. Early surgery in the first 3 years of life would be helpful for the IE patients to have an opportunity to approach the normal development trajectory as early as possible. Otherwise, although they have postoperatively ocular alignment and binocular vision, their functional connectivity in the brain is still abnormal. A previous study indicates that the critical period of binocular vision development begins several months after birth and peaks between 1 and 3 years of age (Banks et al., 1975). Early surgery (within 6 months of onset of infantile esotropia) would be benefit for the infants to establish binocular single vision. Thus, we think that early surgery not only benefit for enhancing the motor and sensory outcomes, but also for establishing functional connectivity in the brain, which in turn to consolidate the motor and sensory outcomes, reducing severity of DVD and minimizing a delay in sensorimotor and gross motor development.

The ORBsup and REC are constituent part of the orbitofrontal cortex (OFC) in the prefrontal lobe (Felix et al., 2021), where the brain processes and transmits various sensory information, including visual, auditory, somatosensory and olfactory information, integrates visceral sensorimotor information, and involves in visuo-affective prediction (Kringelbach, 2005; Chaumon et al., 2014; Peihong et al., 2021). Reduced cortical thickness in OFC has been observed in patients with concomitant strabismus (Yin et al., 2021). A voxel-mirrored homotopic connectivity study shows that interhemispheric functional connectivity between bilateral OFC is reduced in the patients with strabismus and amblyopia (Peng et al., 2021). In this study, the abnormal functional connectivity within OFC indicates that IE patients have a reduced ability in their cortex to process visual information, which is likely caused by asymmetrical visual input from the IE patient’s eyes. Thus, the subcortical visual information processing system is likely to be activated, and to replace the cortical visual information processing system, resulting in infantile esotropia, latent nystagmus and DVD.

The TPOsup is located in the anterior portion of superior temporal gyrus, and sensitive to cross-modal features of object (Ohki et al., 2016). It is involved in auditory-visual information integration and in the process of optokinetic nystagmus (Dieterich et al., 2003; Leminen et al., 2020). Previous studies have shown that there are microstructural and functional changes in the superior temporal gyrus in patients with strabismus (Huang et al., 2016; Tan et al., 2018). The PCG is a part of the oculomotor cortex and plays vital roles in regulating eye movements such as smooth pursuit eye movement and optokinetic nystagmus (Fischer et al., 2012; Ertl et al., 2017; Ruehl et al., 2021). Furthermore, PCG is a high-order hub for incorporating vestibular information and visual motion signals in the perceptual process of self-motion (Ruehl et al., 2021). Recently, a new hypothesis has been proposed to explain the pathogenesis of IE, which is caused by an asymmetric optokinetic response that drives the eyes nasalward. Our finding of abnormal functional connectivity between TPOsup and PCG implies that IE patients may have abnormal optokinetic response and smooth pursuit.

As a crucial component of the visual cortex, MOG plays an important role in visual input integration and processing, involving in the establishment of stereopsis (Zhu et al., 2018). The primary VN communicates with advanced VN through content and spatial pathways, which are regulated by the DMN (Yu et al., 2022). The abnormal functional connectivity between the VN and DMN (i.e., right MOG-left SFGmed) reflects that the visual pathway and stereopsis are damaged in the IE patients.

IOG is the core part of VN, and is able to activate during the analysis of visual features and recognition of facial configuration, which is of great importance for human social interaction (Rossion et al., 2011; Sato et al., 2017). A diffusion tensor imaging study confirmed that IOG shows white matter fiber connections that receive visual input *via* the visual radiation to V1, suggesting its role in visual information processing (Gschwind et al., 2012). The ROL is involved in forming the SMN, which is responsible for motor control. As a vestibular core region, ROL is associated with visual-vestibular interaction, promoting the perception of self-motion and spatial orientation (zu Eulenburg et al., 2012; Helmchen et al., 2020). Aberrant functional connectivity between the VN (i.e., IOG) and SMN (i.e., ROL) in IE patients may interfere the visual-motor integration, which leads to the impaired “hand-eye” coordination. A recent research also demonstrated the abnormal functional connectivity between the VN and SMN in patients with strabismus, which is consistent with our findings (Yu et al., 2022).

The limbic network is tightly associated with emotional processing and cognition function (Jobst et al., 2021; Qiu et al., 2021). Impaired brain functional connectivity caused by strabismus would be possible to lead to emotional and cognitive changes. In fact, it is not uncommon for strabismus patients to experience emotional changes before and after surgery. Recent studies also support the fact that patients with strabismus may present depression or anxiety (Tan et al., 2016; Zhang et al., 2021).

Several limitations should be considered in this study. First of all, although significant group-age interaction was demonstrated by GLM, the trajectory of several functional connections (right PHG-left ORBsup, right ROL-bilateral IOG) with age was not evident in IE group through partial correlation analysis, which may be related to the sampling error. Increasing the sample size can reduce the sampling error and make the results more accurate. In addition, a cross-sectional sample (aged from infancy to early childhood) was used to examine the developmental trajectory of functional connectivity in patients with IE. However, longitudinal studies are important for better understanding how functional connectivity changes over time in IE patients. Furthermore, vision is essential for cognitive and behavioral development. Cognitive assessment should be carried out in future research to determine whether patients with IE are accompanied by cognitive impairment.

## Conclusion

To our knowledge, this is the first study to examine the characteristics of functional connectivity development in IE patients. Abnormal functional network connectivity could present very early after birth in IE patients, and then exhibit aberrant developmental patterns over time. Abnormal functional network in IE patients indicates reduced ability of their cortex in visual information processing, optokinetic nystagmus regulation, “hand-eye” coordination and emotional management. Reduced cortex ability in visual information processing may reactivate subcortical visual information processing system, allowing a nasalward optokinetic bias to drive the eyes inward, resulting in infantile esotropia. The first 3–4 years after birth are critical for IE patients to build a normal functional network in the brain. Early surgery in the first 3–4 years of life would be helpful for the IE patients to have an opportunity to approach the normal development trajectory as early as possible.

## Data availability statement

The original contributions presented in this study are included in the article/Supplementary material, further inquiries can be directed to the corresponding authors.

## Ethics statement

The studies involving human participants were reviewed and approved by the Medical Research Ethics Committee of Beijing Children’s Hospital. Written informed consent to participate in this study was provided by the participants’ legal guardian/next of kin.

## Author contributions

YP, NL, and DH designed the study. JG was in charge of acquiring imaging data and drafting the manuscript. WL, LH, YL, and HK contributed to recruiting participates and collecting clinical data. YC was responsible for data analysis. All authors approved the final manuscript.
