# Supplementary material for: Abnormal developmental trends of functional connectivity in young children with infantile esotropia
Source: Front Neurosci. 2022 Aug 18;16:972882. doi: 10.3389/fnins.2022.972882 (PMC9433796; doi:10.3389/fnins.2022.972882)
Supplement: Supplementary file 1 [file Table_1.DOCX]

**Table S1. Regions of interest included in UNC infant brain atlas**

| Labels | Regions | Regions | Abbreviations |
| --- | --- | --- | --- |
| 1 | Precentral_L | Precentral gyrus | PreCG.L |
| 2 | Precentral_R | Precentral gyrus | PreCG.R |
| 3 | Frontal_Sup_L | Superior frontal gyrus, dorsolateral | SFGdor.L |
| 4 | Frontal_Sup_R | Superior frontal gyrus, dorsolateral | SFGdor.R |
| 5 | Frontal_Sup_Orb_L | Superior frontal gyrus, orbital part | ORBsup.L |
| 6 | Frontal_Sup_Orb_R | Superior frontal gyrus, orbital part | ORBsup.R |
| 7 | Frontal_Mid_L | Middle frontal gyrus | MFG.L |
| 8 | Frontal_Mid_R | Middle frontal gyrus | MFG.R |
| 9 | Frontal_Mid_Orb_L | Middle frontal gyrus, orbital part | ORBmid.L |
| 10 | Frontal_Mid_Orb_R | Middle frontal gyrus, orbital part | ORBmid.R |
| 11 | Frontal_Inf_Oper_L | Inferior frontal gyrus, opercular part | IFGoperc.L |
| 12 | Frontal_Inf_Oper_R | Inferior frontal gyrus, opercular part | IFGoperc.R |
| 13 | Frontal_Inf_Tri_L | Inferior frontal gyrus, triangular part | IFGtriang.L |
| 14 | Frontal_Inf_Tri_R | Inferior frontal gyrus, triangular part | IFGtriang.R |
| 15 | Frontal_Inf_Orb_L | Inferior frontal gyrus, orbital part | ORBinf.L |
| 16 | Frontal_Inf_Orb_R | Inferior frontal gyrus, orbital part | ORBinf.R |
| 17 | Rolandic_Oper_L | Rolandic operculum | ROL.L |
| 18 | Rolandic_Oper_R | Rolandic operculum | ROL.R |
| 19 | Supp_Motor_Area_L | Supplementary motor area | SMA.L |
| 20 | Supp_Motor_Area_R | Supplementary motor area | SMA.R |
| 21 | Olfactory_L | Olfactory cortex | OLF.L |
| 22 | Olfactory_R | Olfactory cortex | OLF.R |
| 23 | Frontal_Sup_Medial_L | Superior frontal gyrus, medial | SFGmed.L |
| 24 | Frontal_Sup_Medial_R | Superior frontal gyrus, medial | SFGmed.R |
| 25 | Frontal_Mid_Orb_L | Superior frontal gyrus, medial orbital | ORBsupmed.L |
| 26 | Frontal_Mid_Orb_R | Superior frontal gyrus, medial orbital | ORBsupmed.R |
| 27 | Rectus_L | Gyrus rectus | REC.L |
| 28 | Rectus_R | Gyrus rectus | REC.R |
| 29 | Insula_L | Insula | INS.L |
| 30 | Insula_R | Insula | INS.R |
| 31 | Cingulum_Ant_L | Anterior cingulate and paracingulate gyri | ACG.L |
| 32 | Cingulum_Ant_R | Anterior cingulate and paracingulate gyri | ACG.R |
| 33 | Cingulum_Mid_L | Median cingulate and paracingulate gyri | DCG.L |
| 34 | Cingulum_Mid_R | Median cingulate and paracingulate gyri | DCG.R |
| 35 | Cingulum_Post_L | Posterior cingulate gyrus | PCG.L |
| 36 | Cingulum_Post_R | Posterior cingulate gyrus | PCG.R |
| 37 | Hippocampus_L | Hippocampus | HIP.L |
| 38 | Hippocampus_R | Hippocampus | HIP.R |
| 39 | ParaHippocampal_L | Parahippocampal gyrus | PHG.L |
| 40 | ParaHippocampal_R | Parahippocampal gyrus | PHG.R |
| 41 | Amygdala_L | Amygdala | AMYG.L |
| 42 | Amygdala_R | Amygdala | AMYG.R |
| 43 | Calcarine_L | Calcarine fissure and surrounding cortex | CAL.L |
| 44 | Calcarine_R | Calcarine fissure and surrounding cortex | CAL.R |
| 45 | Cuneus_L | Cuneus | CUN.L |
| 46 | Cuneus_R | Cuneus | CUN.R |
| 47 | Lingual_L | Lingual gyrus | LING.L |
| 48 | Lingual_R | Lingual gyrus | LING.R |
| 49 | Occipital_Sup_L | Superior occipital gyrus | SOG.L |
| 50 | Occipital_Sup_R | Superior occipital gyrus | SOG.R |
| 51 | Occipital_Mid_L | Middle occipital gyrus | MOG.L |
| 52 | Occipital_Mid_R | Middle occipital gyrus | MOG.R |
| 53 | Occipital_Inf_L | Inferior occipital gyrus | IOG.L |
| 54 | Occipital_Inf_R | Inferior occipital gyrus | IOG.R |
| 55 | Fusiform_L | Fusiform gyrus | FFG.L |
| 56 | Fusiform_R | Fusiform gyrus | FFG.R |
| 57 | Postcentral_L | Postcentral gyrus | PoCG.L |
| 58 | Postcentral_R | Postcentral gyrus | PoCG.R |
| 59 | Parietal_Sup_L | Superior parietal gyrus | SPG.L |
| 60 | Parietal_Sup_R | Superior parietal gyrus | SPG.R |
| 61 | Parietal_Inf_L | Inferior parietal, but supramarginal and angular gyri | IPL.L |
| 62 | Parietal_Inf_R | Inferior parietal, but supramarginal and angular gyri | IPL.R |
| 63 | SupraMarginal_L | Supramarginal gyrus | SMG.L |
| 64 | SupraMarginal_R | Supramarginal gyrus | SMG.R |
| 65 | Angular_L | Angular gyrus | ANG.L |
| 66 | Angular_R | Angular gyrus | ANG.R |
| 67 | Precuneus_L | Precuneus | PCUN.L |
| 68 | Precuneus_R | Precuneus | PCUN.R |
| 69 | Paracentral_Lobule_L | Paracentral lobule | PCL.L |
| 70 | Paracentral_Lobule_R | Paracentral lobule | PCL.R |
| 71 | Caudate_L | Caudate nucleus | CAU.L |
| 72 | Caudate_R | Caudate nucleus | CAU.R |
| 73 | Putamen_L | Lenticular nucleus, putamen | PUT.L |
| 74 | Putamen_R | Lenticular nucleus, putamen | PUT.R |
| 75 | Pallidum_L | Lenticular nucleus, pallidum | PAL.L |
| 76 | Pallidum_R | Lenticular nucleus, pallidum | PAL.R |
| 77 | Thalamus_L | Thalamus | THA.L |
| 78 | Thalamus_R | Thalamus | THA.R |
| 79 | Heschl_L | Heschl gyrus | HES.L |
| 80 | Heschl_R | Heschl gyrus | HES.R |
| 81 | Temporal_Sup_L | Superior temporal gyrus | STG.L |
| 82 | Temporal_Sup_R | Superior temporal gyrus | STG.R |
| 83 | Temporal_Pole_Sup_L | Temporal pole: superior temporal gyrus | TPOsup.L |
| 84 | Temporal_Pole_Sup_R | Temporal pole: superior temporal gyrus | TPOsup.R |
| 85 | Temporal_Mid_L | Middle temporal gyrus | MTG.L |
| 86 | Temporal_Mid_R | Middle temporal gyrus | MTG.R |
| 87 | Temporal_Pole_Mid_L | Temporal pole: middle temporal gyrus | TPOmid.L |
| 88 | Temporal_Pole_Mid_R | Temporal pole: middle temporal gyrus | TPOmid.R |
| 89 | Temporal_Inf_L | Inferior temporal gyrus | ITG.L |
| 90 | Temporal_Inf_R | Inferior temporal gyrus | ITG.R |
